# Molecular Identification of *Ancylostoma caninum* Isolated from Cats in Southern China Based on Complete ITS Sequence

**DOI:** 10.1155/2013/868050

**Published:** 2013-09-23

**Authors:** Yuanjia Liu, Guochao Zheng, Muhamd Alsarakibi, Xinheng Zhang, Wei Hu, Pengyun Lu, Liqin Lin, Liping Tan, Qin Luo, Guoqing Li

**Affiliations:** ^1^College of Veterinary Medicine, South China Agricultural University, Guangzhou 510642, China; ^2^College of Animal Science, South China Agricultural University, Guangzhou 510642, China

## Abstract

*Ancylostoma caninum* is a blood-feeding parasitic intestinal nematode which infects dogs, cats, and other mammals throughout the world. A highly sensitive and species-specific PCR-RFLP technique was utilised to detect the prevalence of *A. caninum* in cats in Guangzhou, southern China. Of the 102 fecal samples examined, the prevalence of *A. caninum* in cats was 95.1% and 83.3% using PCR-RFLP and microscopy, respectively. Among them, the prevalence of single hookworm infection with *A. caninum* was 54.90%, while mixed infections with both *A. caninum* and *A. ceylanicum* were 40.20%. Comparative analysis of three complete ITS sequences obtained from cat-derived *A. caninum* showed the same length (738 bp) as that of dog-derived *A. caninum*. However, the sequence variation range was 98.6%–100%, where only one cat isolate (M63) showed 100% sequence similarity in comparison with two dog-derived *A. caninum* isolates (AM850106, EU159416) in the same studied area. The phylogenetic tree revealed *A. caninum* derived from both cats and dogs in single cluster. Results suggest that cats could be the main host of *A. caninum* in China, which may cause cross-infection between dogs and cats in the same area.

## 1. Introduction

*Ancylostoma caninum* is a blood-feeding parasitic intestinal nematode which infects dogs, cats, and other mammals throughout the temperate and tropical areas in the world [1–3]. In addition to the veterinary importance, *A. caninum* can also cause zoonotic disease in humans. The larvae of *A. caninum* hatch from eggs and develop into infective larvae via two molts. The infective larvae then infect host animals such as dogs and cats, migrate into the intestine, and develop into adult worms following two more molts. If the infective larvae invade humans, they can cause cutaneous larvae migrans (CLM) or “creeping eruptions,” which are hypersensitive reactions in response to the migration of *A. caninum* larvae; however, they cannot develop into adult worms just by migrating under the skin [4].

Although some cases recorded that *A. caninum* was found in cats [5–8], this species has been still regarded as an uncommon parasite of cats. Therefore, *A. caninum* was described as “dog hookworm” [9] and was supposed as a host-specific parasite for canids [2, 10], while Palmer stated that *A. caninum* was the predominant species of hookworm in dogs [11], and *A. tubaeforme* was the predominant species of hookworm in cats. In China, high occurrence of *A. caninum* has been reported with prevalence of 1.04%–73%, but without significant area differences [12, 13]. Although *A. caninum* in cats has been reported in Thailand (23%) [14], Australia (30%) [11], and Sichuan province in China (25% and 51%) [15, 16], data on prevalence of *A. caninum* in cats are still scarce.

Herein, this study presents the first molecular identification based on complete ITS sequence, as well as it describes a simple and effective detection method for *A. caninum* from cats in southern China.

## 2. Materials and Methods

### 2.1. Area Studied

Guangzhou city is located in south-central Guangdong province (N: 22°45′~23°05′; E: 113°14′~113°34′), southern China, experiencing a typically tropical climate with heavy monsoon rains. It covers an area of approximately 8000 km^2^, divided into 10 geographical districts with an estimated population of approximately 12 million. This city contains a large number of sheltered cats and owns two local humane shelters for stray cats in Conghua and Baiyun districts.

### 2.2. Fecal Sample Collection and Processing

Cat fecal samples (*n* = 102) were collected from Conghua (*n* = 72) and Baiyun (*n* = 30) humane shelters during March and July 2012. A single sample was collected in clean container from each cat, directly transported to laboratory, preserved in 2.5% potassium dichromate, and stored at 4°C. To detect the presence of hookworm eggs, direct microscopic examination was done by saturated sodium chloride and glucose flotation. Positive fecal samples were further characterized by molecular procedures.

### 2.3. Genomic DNA Extraction

DNAs were extracted directly from fecal samples using a commercial DNA extraction kit (QIAamp DNA Stool Mini Kit, QIAgen, Hilden, Germany) according to the manufacturer's protocols. However, all samples were pretreated with 5 cycles of heating at 100°C for 5 minutes, followed by immediate freezing at −80°C for 5 minutes. A negative control (water) was used in each extraction group. Extracted DNAs were then stored at −20°C.

### 2.4. Primers and Restriction Enzyme

One pair of primers, AF (5′-CTTTGTCGGGAAGGTTGG-3′) and AR (5′-TTCACCACTCTAAGCGTCT-3′), was designed from conserved region of ITS sequences of five species of hookworms including *A. caninum* (AM85010, AM850105), *A. tubaeforme* (JQ812691), *A. ceylanicum* (DQ381541, DQ780009), *A. braziliense* (DQ359149, DQ438056), and *U. stenocephala* (HQ262053, AF194145) by Primer Premier 5.0 to amplify 404 bp region of *A. caninum,* 405 bp of *A. tubaeforme*, 408 bp of *A. braziliense,* 404 bp of *A. ceylanicum*, and 406 bp of *U. stenocephala,* which contain ITS1 and 5.8S rRNA regions. The five species of hookworms could be distinguished by restriction endonucleases *Eco*RII, *Bsu*RI, and *Taq* I for different cutting sites on the sequence according to the analysis by Primer Premier 5.0. The theoretical cutting patterns of the five different hookworm DNA fragments treated by three restriction enzymes are shown in Table 1. *EcoR* II could identify *A. ceylanicum* and *A. braziliense*, while *Bsu*RI can only identify *U. stenocephala*, and *Taq* I was distinguished between *A. caninum* and *A. tubaeforme*.

Another pair of primers CAF (5′-GACTGCGGACTGCTGTAT-3′) and CAR (5′-AAGTTCAGCGGGTAGTCA-3′) was designed by Primer Premier 5.0 based on ITS sequences (JQ812694, AJ920347, and AM039739) of *A. caninum* to amplify the complete ITS sequence of cat-derived *A. caninum*.

### 2.5. PCR-RFLP

Both PCRs were performed in 25 *μ*L volume containing 2 *μ*L of the DNA sample, 0.2 *μ*L of *Taq* polymerase (TaKaRa, Dalian, China), 2.5 *μ*L of 10×*Taq* buffer (TaKaRa), 2 *μ*L of diethylnitrophenyl thiophosphate (dNTP, TaKaRa) mixture, 0.5 *μ*L of each primer (AF/AR or CAF/CAR, 50 mM), and 17.3 *μ*L of distilled water. PCR cycling parameters were as follows: 1 cycle at 96°C for 5 minutes, then 35 cycles of 96°C for 30 seconds, at 60°C for 30 seconds, and at 72°C for 90 seconds, followed by 1 cycle at 72°C for 7 minutes.

RFLP analysis was performed by digesting 7 *μ*L of PCR product with 2 U of each restriction endonuclease (TaKaRa) in a final volume of 20 *μ*L for 3 hours at 37°C. PCR products and restriction fragments were analyzed after electrophoresis in 2% and 3% agarose gels with 0.2 *μ*g/mL of ethidium bromide staining and were visualized on a UV transilluminator.

### 2.6. Sequence Confirmation and Phylogenetic Analysis

Positive amplicons were purified and sequenced using ABI 3730 automated DNA sequencer (BigDye Terminator Chemistry). Obtained sequences were aligned with 15 ITS reference sequences using Clustal X programs [17]. Phylogenetic trees were constructed using MEGA version 5.1 (MEGA5.1: Molecular Evolutionary Genetics Analysis software, Arizona State University, Tempe, Arizona, USA). Bootstrap analyses were conducted using 1,000 replicates to assess the reliability of inferred tree topologies. Neighbor-joining algorithms were conducted using the Kimura 2 parameter distance analysis. Obtained nucleotide sequences have been deposited in the GenBank database under accession numbers KC755015 and KC755025.

## 3. Results

Of the 102 collected fecal samples, 85 samples (83.3%) were microscopically positive, while 97 samples (95.1%) were PCR positive for hookworm. The prevalence of *A. caninum* in cats from suburban area (86.1%, Conghua) was higher than that from urban area (76.7%, Baiyun). 

**Figure 1:**
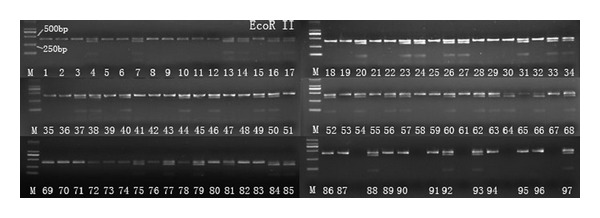
PCR amplicons digested by restriction endonucleases *EcoR *II. Lanes 1–62: samples from Conghua district; lanes 62–85: samples from Baiyun district; lanes 86–97: microscopically negative samples; M: DL-2000 DNA marker.

**Figure 2:**
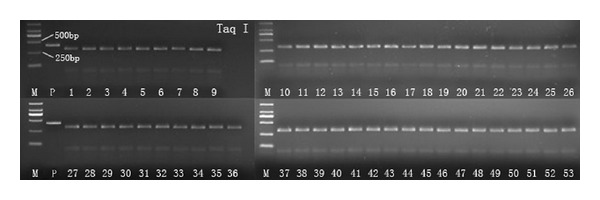
PCR amplicons digested by restriction endonucleases *Taq* I. Lanes 1–53: random samples from 56 samples; P: PCR amplicon; M: DL-2000 DNA marker.

**Figure 3:**
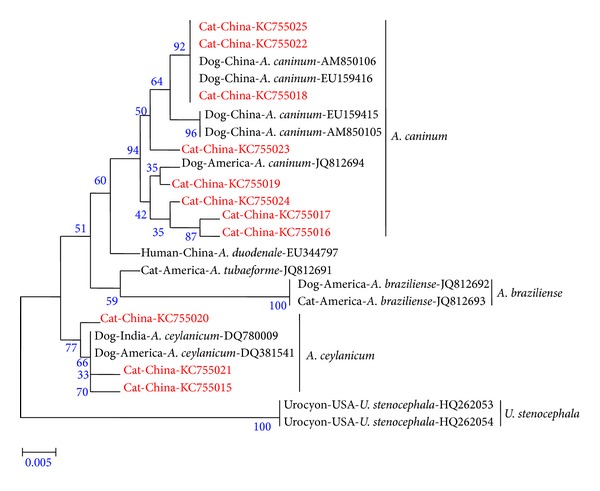
Phylogenetic tree of hookworm isolates based on the ITS1 and 5.8S rRNA sequences with neighbor-joining algorithm using Kimura two-parameter. The reference sequences are available in the GenBank by their accession numbers. The isolates of this study are shown in red color.

The results showed that *U. stenocephala* was absent in the 97 examined PCR samples, where there was no enzymatic digestion by restriction endonucleases *Bsu*RI (not shown).  Figure 1 shows the digestion results of the 97 PCR amplicons by restriction endonucleases *EcoR* II, in which 41 samples showed three bands, revealing that these samples were mixed infections with *A. ceylanicum* and one undetermined hookworm (*A. caninum *or other), while 56 samples showed one band, revealing that these samples were single infection with one undetermined hookworm (*A. caninum *or other). Fifty three samples from the 56 single infection samples were randomly chosen for the *Taq* I digestion; the results show that all these samples were infected with only one species (Figure 2). Thereafter, 11 positive samples (6/56 and 5/41) were successfully sequenced; the phylogenetic tree based on those sequences with 15 reference sequences (Figure 3) revealed that the 56 positive samples were infected with *A. caninum*, while the 41 samples were infected with both *A. ceylanicum* and *A. caninum*, without any *A. tubaeforme* infection in our study. Thus, the overall prevalence of *A. caninum* infection was 95.1%, with a prevalence of 54.9% single infection and 40.2% mixed infections with *A. ceylanicum*.

Three complete ITS sequences from cat-derived *A. caninum* isolates (M45, M63, and M84) were obtained by Primer CAF/CAR and submitted in the GenBank under accession numbers (KC755026, KC755028, and KC755029). All ITS sequences were 738 bp in length, which was the same length presented by the *A. caninum* ITS region (AM850105, AM850106, EU159415, and EU159416) isolated from dogs in this area. DNA sequences were assembled using DNAStar (version 7; Madison, WI, USA) and multiple-sequence alignment was performed with MegAlign program. Comparative analysis of the three complete ITS sequences obtained from cat-derived *A. caninum* showed the same length (738 bp) of dog-derived* A. caninum*, with a similarity of 99.2%–99.7%. However, compared to the ITS sequences of dog-derived *A. caninum* isolates (AM850105, AM850106, EU159415, and EU159416), the sequence variation range was 98.6%–100%, where only one cat isolate (M63) showed 100% sequence similarity compared with two dog-derived *A. caninum* isolates (AM850106, and EU159416) in the same studied area. The sequence similarities of the cat-derived *A. caninum* with *A. ceylanicum* (KC755027), *A. tubaeforme* (JQ812691), and *A. braziliense* (JQ812692) were 97.0%–97.3%, 97.3%–97.6%, and 91.6–92.1%, respectively.

## 4. Discussion

As early as the 19th century, hookworms in cats and dogs had been described by Zedler (1800) and Ercolani (1859) [31]. For nearly 100 years, the common hookworm of both dogs and cats was referred as *Ancylostoma caninum* [32]. Later then, some authors stated that it was difficult to infect dogs with larvae from cats, and vice versa [32–36]. Studies in Europe, Africa, and Australia described the morphological differences of hookworms obtained from dogs and cats, where *A. caninum* was distinguished from *A. tubaeforme* and was thought to be host-specific for dogs [31, 32].

To date, feline hookworms (including *A. tubaeforme*, *A. braziliense*, *A. ceylanicum*,* A. caninum*,and *U. stenocephala*) had been reported nineteen times worldwide (Table 2). Obviously, *A. tubaeforme *was the most common species which was reported in Australia (6 times) [6, 7, 11, 19, 21, 22], USA (2 times) [29, 30], once in South America (Brazil) [23], Central America (Costa Rica) [24], Europe (Italy, Spain) [25, 28], and Middle East (Qatar) as well [27], while this parasite had not been reported from cats in China (East Asia) [15, 16], Malaysia (Southeast Asia) [26], and Thailand [14].

In China, *A. caninum* was reported twice in Sichuan province, southwest China, with prevalence of 25% and 51%, respectively, [15, 16], while in our survey, overall higher prevalence (95.1%) of *A. caninum* infections in cats was detected in Guangzhou (southern China). Our results suggested that the predominant species of hookworms in cats was *A. caninum* in China, while *A. tubaeforme* was considered to be the predominant species in Australia [11], which strongly supports our suggestion that the prevalent species is related to its geographical distribution, as well as cats could be the main host for *A. caninum* in China.

The first cat-derived *A. caninum* complete ITS sequences (GenBank: KC755026, KC755028, and KC755029) are presented in our study. The length of obtained sequences (738 bp) was identical to the dog-derived *A. caninum* ITS sequence, as well as the sequence similarity range was 98.6%–100%. In addition, *A. caninum* from both cats and dogs was connected in a single cluster in the constructed phylogenetic tree. This finding indicated that there could be a cross-infection of *A. caninum* between dogs and cats in the studied areas. 

In conclusion, the results of this study demonstrated that The PCR-RFLP technique described in this study was a rapid and straightforward method for the identification and discrimination of *A. caninum.* Moreover, the ITS sequences could be used to identify this hookworm species from different local hosts. Current information regarding the prevalence of *A. caninum* showed possible cross-infections between different hosts. Therefore, it is imperative to have current information regarding the prevalence of this hookworm and the associated risk factors of this infection. This will allow a more effective implementation of strategic control programmes for hookworm infections.

## Figures and Tables

**Table 1:** Predictive restriction patterns by endonucleases *EcoR* II, *BsuR* I, and *Taq* I at ITS1 and 5.8S locus.

Species	PCR amplicon (bp)	Cleavage site			Predicted fragment size (bp)
		*Eco*R II	*Bsu*R I	*Taq* I	
*A. caninum *	404	−	−	3+	60,307 (12^U^, 25^U^)
*A. tubaeforme *	405	−	−	2+	60,333 (12^U^)
*A. ceylanicum *	404	+	−		76,328
*A. braziliense *	408	2+	−		76,122,210
*U. stenocephala *	406	−	+		87,319

^U^Means that the fragment is too small to visible.

**Table 2:** Previous investigations of hookworm species in cats worldwide.

Country (area)	Detection method	Species	Prevalence	Source of animal	Reference
Australia (Sydney)	Necropsy	*A. tubaeforme *	35%	Pound	[6]
		*U. stenocephala *	0.5% (*n* = 404)		
Australia (Tasmania)	Necropsy	*U. stenocephala *	2% (*n* = 86)	Feral cats	[18]
Australia (Brsibane)	Necropsy	*A. tubaeforme *	19% (*n* = 404)	Pound	[7]
Australia (Brisbane)	Necropsy	*A. tubaeforme *	81%	Pound	[19]
Australia (Adelaide)	Fecal exam	*U. stenocephala *	0.3% (*n* = 376)	Vet practice	[20]
Australia (Kimberley)	Necropsy	*A. tubaeforme *	20% (*n* = 34)	Aboriginal community	[21]
Australia (Northern territory)	Necropsy	*A. tubaeforme *	13%	Feral cats	[22]
Australia (countrywide)	PCR-RFLP	*A. tubaeforme *	70% (*n* = 10)	Refuge and pet cats	[11]
		*A. caninum *	30% (*n* = 10)		
Brazil (Rio de Janeiro)	Necropsy	*A. braziliense *	65.9% (*n* = 135)	Cat (shelters and Zoonoses Control Center)	[23]
		*A. tubaeforme *	8.9% (*n* = 135)		
China (Sichuan)	Necropsy	*A. caninum *	25% (*n* = 20)	Domestic cat	[15]
		*A. braziliense *	10% (*n* = 20)		
		*A. ceylanicum *	5% (*n* = 20)		
		*U. stenocephala *	10% (*n* = 20)		
China (Sichuan)	Necropsy	*A. caninum *	51% (*n* = 149)	Domestic cat	[16]
		*A. braziliense *	17.4% (*n* = 149)		
		*U. stenocephala *	14% (*n* = 149)		
Costa Rica (San Isidro EL. General)	Fecal exam	*A. tubaeforme *	1.1% (*n* = 9)	Refuge cat	[24]
Thailand (Prachin Buri)		*A. ceylanicum* *A. caninum*	92% 23%	Cat	[14]
Italy (central)	Larvae (L3) exam	*A. tubaeforme *	1.2% (*n* = 81)	Cat (pet)	[25]
		*U. stenocephala *	3.7% (*n* = 81)		
Malaysia (West Malaysia)	PCR-sequencing	*A. ceylanicum* *A. braziliense*	26.1% (*n* = 23) 4.3% (*n* = 23)	Domestic cat	[26]
Qatar (Doha)	Necropsy	*A. tubaeforme *	14.7% (*n* = 658)	Cat (feral)	[27]
Spain (mid-Ebro Valley)	Necropsy	*A. tubaeforme *	29.3% (*n* = 58)	Cat (stray)	[28]
USA (Florida)	Necropsy	*A. tubaeforme *	75% (*n* = 60)	Cat (feral)	[29]
		*A. braziliense *	33% (*n* = 60)		
USA (Pennsylvania)	Fecal exam	*A. tubaeforme *	0.5% (*n* = 1566)	Cat (pet)	[30]
